# Clinical nursing education during the COVID-19 pandemic: perspectives of students and clinical educators

**DOI:** 10.1186/s12912-022-01029-3

**Published:** 2022-10-26

**Authors:** Omolhoda Kaveh, Fahimeh Ghasemi Charati, Mahsa Kamali, Fereshteh Araghian Mojarrad

**Affiliations:** 1 School of Nursing and Midwifery Sari, Mazandaran University of Medical Sciences, Sari, Iran; 2 Department of Medical Surgical Nursing, Nasibeh Faculty of Nursing and Midwifery, Mazandaran University of Medical Sciences, Sari, Iran; 3 Pediatric Infectious Diseases Research Center, Communicable Disease Institute, Mazandaran University of Medical Sciences, Sari, Iran; 4 Research Center for Traditional and Complementary Medicine, Mazandaran University of Medical Sciences, Sari, Iran

**Keywords:** Clinical education, Nursing, COVID-19, Qualitative study

## Abstract

**Background:**

The outbreak of the COVID-19 pandemic has thus far disrupted clinical nursing education (CNE) around the world and in Iran; which has encountered numerous challenges to this point for students and clinical educators. Therefore, this qualitative study was conducted to identify the perspectives of nursing students and clinical educators during the COVID-19 pandemic.

**Methods:**

In this qualitative descriptive study, 15 participants, including nursing students and clinical educators, were selected through the purposive sampling method. The data were collected from March to September 2021 in nursing school through in-depth semi-structured interviews, and then analyzed based on the Graneheim and Lundman method.

**Results:**

Two main themes, “*CNE in the shadows of threats and opportunities*” and “*CNE recovery*” and five themes (*Injured CNE, CNE under fear, Lessons from the COVID-19 crisis, Providing optimal CNE by eradicating COVID-19*, and *Adapting CNE to existing conditions*) were extracted from the data analysis.

**Conclusions:**

The results of this qualitative study demonstrated that the COVID-19 pandemic had led the participants to experience new conditions, also referred to as “combined threats and opportunities”. Based on this, nursing managers and planners are advised to take great steps towards the development and improvement of CNE by considering and applying these results in their plans to overcome the challenge of CNE with effective and useful actions in the COVID-19 pandemic.

## Background

The “stay-at-home” recommendation was established on March 19, 2020, to possibly prevent the spread of the coronavirus disease [1]. The world is also now drawn against an unprecedented challenge caused by this pandemic that has so far claimed thousands of lives [2]. The workload imposed on healthcare systems has also led to global concerns, and such crises have affected not only frontline healthcare workers and clinical leadership but also entire healthcare systems and communities [3, 4].

COVID-19 has further interrupted the activities of universities and educational institutions and thus burdened the workloads of faculties and educational institutions. In the field of healthcare services, the schools of nursing have to prepare to deal with unique challenges related to their roles in developing the next generation of healthcare workers [5]. The rapid expansion of COVID-19, however, has severely hindered the ability of medical and nursing students to engage with patients and healthcare professionals [6]. Issues with the quality of medical education have arisen as a result of the clinical practicum’s passive role. As a result, in order to fulfill their purpose of developing capable future healthcare professionals, the faculty instructors had to quickly address the holes in the clinical practicum [7]. Serious concerns related to learner safety were at the forefront. To ensure their own and students’ safety, academic institutions, like many other professions, have been forced to move all classes, meetings, and face-to-face discussions into virtual environments [8]. Therefore, the schools of nursing are expected to be updated with the latest teaching strategies and technologies to demonstrate their leadership and management skills and, provide high-quality methods to foster students and ultimately their affiliated organizations [9, 10].

Among the challenges to medical educational systems around the world is the transfer of appropriate clinical experiences to students in terms of dealing with patients and working in hospital settings, which cannot be fulfilled through virtual and online education, like theoretical courses [11].

To date, numerous studies have been conducted on the challenges of CNE under normal circumstances, such as scientific, practical, and emotional weaknesses in clinical instructors; low knowledge and motivation among students; inadequate bedside facilities and equipment; and unfitting cooperation between hospital and nursing staff; educators and students, poor management and planning; and no harmony between departments [5, 8, 12, 13]. Clinical education is comprised of different actors such as educators, students, staff, and management, each one as one part of the pyramid, whose integration can create more effective and desirable training [12].

Clinical education also forms the basis of nursing education and allows students to apply their theoretical knowledge to care delivery for patients in different wards [14]. In fact, clinical education represents a process in which students attend the patient’s bedside, gradually gain enough experience, and prepare themselves using logical thinking to deal with patient problems [15]. This type of education is therefore crucial for shaping their professional identity to the extent that the formation of such an identity depends on the type and manner of education and their role in medical settings. However, the COVID-19 crisis, with its special circumstances, has posed new challenges to be added to the existing ones; so, further considerations are needed [7, 13]. Experiences in other countries also show that students have failed to play a role in diagnosing and treating patients despite their presence in hospital wards at the onset of this pandemic because there has been insufficient personal protective equipment. With the worsening disease progression, schools have also gradually exempted students from attending clinical wards and considered it necessary to adopt educational schemes based on maintaining student safety and engaging them in remote patient monitoring. In such a situation, the various methods of transferring experience and training to students during the pandemic are constantly evolving, and some alternatives have been raised, including holding online classes only to prepare students to enter hospital wards after the normalization of the conditions, using simulated virtual cases and clinical scenarios; making changes in academic calendars; replacing educational activities with research work (such as papers and dissertations, etc.) as well as adjusting the time of rotations; and encouraging students to get involved in voluntary activities such as the provision of virtual health care and counseling services; and participation in elective courses [1, 16, 17]. In this respect, Mehrdad et al. in Iran designed web-based teaching methods in the form of a clinical teaching model for clinical educators in this pandemic [18]. According to the findings of a systematic review, more research should be conducted to design quasi-experimental studies to evaluate the efficacy of virtual learning training interventions for distance nursing education so that nurses are fully prepared for emergency situations [19].

In order to help the general public and their clinical colleagues, nurse educators will need to make difficult judgments. The requirements of existing and upcoming nursing students must, however, also be taken into account [5].

Although research has thus far identified the challenges facing CNE; no study, to the best of the authors’ knowledge, has addressed the challenges posed by the COVID-19 era, due to the changes in teaching methods in Iran. Given the above-mentioned reasons and the importance of clinical education to nursing students, along with the uncertain time of eradicating such a pandemic, this qualitative study was conducted with the aim of identifying the perspectives of students and clinical educators during the COVID-19 pandemic to help adopt appropriate measures to promote desirable and effective training.

## Methods

### Study design

This descriptive qualitative study was conducted from March 2021 to September 2021. The qualitative description is an outstanding methodological study due to its supply of rich and valuable descriptive content from the subjects’ perspectives [20].

### Participants and setting

The study population consists of nursing students and clinical educators that were selected through the purposive sampling method in nursing school. Purposive sampling is a technique widely used in qualitative research to identify and select participants who have rich experience with the phenomenon of interest [21, 22]. This strategy is designed to increase understanding of selected individual experiences while developing concepts and theories [23]. In accordance with qualitative research, sampling was started with purposeful sampling and continued with maximum variation. In purposeful sampling, the researcher is looking for those who have a rich experience of the phenomena under study and have the ability and desire to express them. The inclusion criteria for the study were having at least one semester of at least one internship semester during COVID-19, clinical educators with a history of CNE at the time of the pandemic, and a willingness to express their own experiences. Exclusion criteria were self-report of the presence of other physical or mental illness; or lack of interest in continuing to participate in the study. This study tried to cover a sample with great variety in age, gender, marital status, semester, and educators with various backgrounds in CNE.

### Data collection

In-depth semi-structured interviews were used for data collection. Upon selecting a quiet and appropriate room based on the participants’ consent (clinical educators’ room in the hospital or faculty) to conduct the interviews; the participants were selected upon meeting the inclusion and exclusion criteria. After obtaining informed consent from the participants and assuring them about the confidentiality of the information, face-to-face interviews using open-ended questions were further conducted by one researcher (Ph.D. in nursing). The interviews were also recorded with the participants’ consent. During the interviews, open-ended questions were additionally raised to allow the participants to put their experiences into plain words freely. Some examples of the questions addressed to the clinical educators were: “*Can you tell me about one day of internship during COVID-19*?”; “*What challenges did you encounter in the process of clinical education*?”; “*What are the facilitators of* CNE *in such conditions*?”; “*What issues and problems did you face during* CNE?”; and “*What did you do to improve the internship process and deal with challenges and problems*?”. As well, some examples of the questions addressed to the students were: “*Can you please describe one day of your internship during COVID-19*?”; “*What did make your internship better during the pandemic*?; and “*What did disturb your internship in such conditions*?*” “Please explain*”. Some follow-up and exploratory questions were also posed based on the data provided by the participants to clarify the concepts and deepen the interview process. Sampling was further continued until data saturation was reached to the extent that no new data was extracted as the interviews continued. The researcher attempted to be an active listener as much as possible during the interviews. The average interview time also lasted 30-60 minutes, depending on the participants. No interview was repeated.

### Data analysis

We used a content analysis approach to analyze the data. Qualitative content analysis is a research approach that is well-suited to analyzing multi-dimensional nursing phenomena and it is for the interpretation of the content of text through coding and identifying themes and patterns [24, 25]. According to the Graneheim and Lundman qualitative method, which consists of five phases [26], the author first attentively listened to the interview recordings numerous times and then transcribed them. To ensure that the interviews were correctly transcribed, author 2 listened to the recordings again and rectified any errors in the transcriptions. Two authors studied the text of the interviews several times in the second stage to gain a complete understanding of the content. In the third stage, the sentences were carefully checked line by line, and the starting codes were recovered. In the fourth phase, all the interviews were thus considered as a unit of analysis. Paragraphs, sentences, or words were accordingly regarded as semantic units. According to their embedded concepts, the semantic units then reached the level of abstraction and conceptualization and were coded. The codes were also compared with each other in terms of their similarities and differences, and they were classified under abstract themes with a specific label. Ultimately, in the fifth phase, by comparing the categories with each other and carefully in-depth reflections on them, the content hidden in the data was introduced as the subject of the study, and the themes were retrieved at the end.

### Trustworthiness

For rigor, the criteria proposed by Guba and Lincoln (credibility, dependability, confirmability, and transferability) were exploited [27]. The researcher accordingly tried to augment the credibility of the study by: long-term engagement, adequate interactions, and good relationships with the participants; devoting enough time to valid information gathering; confirmation of the participants’ information; and getting feedback from the participants. In the review by colleagues, the coding and classification processes were reviewed by other members of the group, and in cases where there was disagreement, discussions were held until an agreement was reached. The systematic repetition, data collection, and analysis using expert reviews (2nd Ph.D. in nursing) were also done to boost data dependability. To assess the dependability, the study process was described in detail and research notes were carefully written. In order to increase data confirmability, the approval of two experts in the field of qualitative research and their supplementary comments were used. The transferability of the study data was further achieved by providing a rich description of the report (environment, participants, non-verbal behaviors, demographic characteristics of the participants, and varied sampling) and the applicability of the research in other fields, so that other researchers could be able to understand the data and access them similarly, and then the participants’ quotes were presented as stated. The checklist of COREQ qualitative designs was used to assess this study [28].

## Results

The study participants consisted of 15 cases, including nine women (60%), with an age range of 20-58 (35.40 ± 1.36) (Table 1). Based on the in-depth interview outcomes and the rich descriptions by the participants, 580 initial codes were extracted. After reviewing the codes several times, they were summarized and categorized with reference to their similarities. Using analyses and comparisons, the hidden content of the codes was obtained, and they were placed into 25 sub-themes and 12 themes, and then named in a conceptual and abstract manner. Finally, 2 main themes, 5 themes, and 17 subthemes were achieved (Table 2).

**Table 1:** Demographic characteristics of the study participants

Participant	Position	Age (years old)	Gender	Marital status	Semester	Work experience (years)
1	Clinical educator (Master’s in nursing)	42	Female	Married	–	20
2	Clinical educator (Master’s in nursing)	44	Female	Married	–	22
3	Student	21	Male	Single	5	–
4	Clinical educator (PhD in nursing)	43	Female	Single	–	18
5	Student	21	Male	Single	7	–
6	Student	22	Female	Single	8	–
7	Clinical educator (PhD in nursing)	46	Female	Married	–	22
8	Student	35	Female	Married	8	–
9	Clinical educator (PhD in nursing)	38	Male	Married	–	5
10	Student	21	Male	Single	8	–
11	Student	20	Male	Single	6	–
12	Clinical educator (PhD in nursing)	58	Male	Married	–	28
13	Student	20	Female	Single	4	–
14	Clinical educator (PhD in nursing)	53	Female	Married	–	24
15	Clinical educator (Master’s in nursing)	40	Female	Married	–	5

**Table 2:** Main themes, themes and sub- themes

Main theme	themes	Sub-themes
CNE in the shadows of threats and opportunities	Injured CNE	Lack of learning opportunities
		Inadequate planning
		Unsatisfactory skill acquisition
	CNE under fear	Fear of COVID-19
		Fear of being infected with COVID-19
		Fear of transmitting COVID-19 to family
		Experience of numerous fears during internship courses
		Intimidating care
	Lesson from the COVID-19 crisis	Personal and professional lifestyle changes among students and educators
		Experience of using other teaching methods by educators
		Experience in integrating virtual teaching with face-to-face internship courses
CNE recovery	Providing optimal CNE by eradicating COVID-19	Establishing interdisciplinary interactions to eradicate COVID-19
		Benefiting from experiences in other countries
		Being vaccinated for COVID-19
	Adapting CNE to existing conditions	Benefiting from experiences of Universities around the world
		Using all potentials available
		Empowering clinical educators

### CNE in the shadows of threats and opportunities

The study participants underlined that CNE was accompanied by some threats and opportunities for educators and students. In this regard, the themes emerging from this main theme were injured CNE, CNE in fear, and lessons from the COVID-19 crisis.

#### Injured CNE

The study results revealed that the quality of CNE had faced shortcomings for some reasons, such as the lack of learning opportunities, inadequate planning, and unsatisfactory skill acquisition. Due to the COVID-19 pandemic and the allocation of most hospital wards to infected patients, short internship courses, packed wards due to the overcrowding of physicians, students, and patients, the non-hospitalization of elective patients, and some changes in daily-use educational departments have thus diminished learning opportunities for students. For example, participant no. 3 said that:

“*At this time, most hospital wards have been dedicated to COVID-19 and there is only one ward that can provide students with clinical education*.”

Participant no. 1 also reiterated that:

“*The ward is so crowded in the morning. Various patients in need of different services are hospitalized in the ward. It is not even possible to walk in it.*”

As well, according to participant no. 10:

“*Two years ago, when the students attended the emergency room for three weeks, they failed to see many cases of multiple traumas, suicide, and poisoning. These cases are now very low.*”

The study findings also showed that the clinical plans had altered due to some changes in the use of hospital wards; no educational space; inadequate internship planning; the elimination of clinical competency exams; and inadequate knowledge among educators about the real number of students. For example, participant no. 7 stated that:

“*The internship schedule is not still clear. It always changes. Every day, when I come to the patient’s bedside, I have to first ask the hospital nursing office about the education department. This makes me feel weak.*”

Moreover, as added by participant no. 6:

“*As a clinical educator, I do not exactly know how many students are going to start the internship because there are five people in the program but only three cases will attend.*”

Due to the major changes in CNE, the participants’ experiences also indicated their limited skill acquisition so that they had learned some abilities to perform procedures with difficulty or that they were not able to fulfill nursing practices independently. They had not acquired the necessary skills due to their fears of contracting and transmitting the disease and even had not given much importance to obtaining a nursing history as a basic principle of patient care. Frequent breaks occurring in CNE due to some changes in the region and the country have further caused these skills to be forgotten. On the other hand, there was an inconsistency between theoretical and clinical courses due to inadequate equipment. For example, according to participant no. 9:

“*Approximately 90-95% of the time, the relationship with the patient is as low as it was before the pandemic.*”

Moreover, participant no. 5 reiterated that:

“*There is fear in case someone might be a virus carrier and such things. As well, taking a history here was canceled, so we did not acquire any new skills.*”

Participant no. 12 also said that:

“*At the school of nursing, the students learn about a series of things academically, such as when and how to wash their hands…. Well, when students come to the wards, they see that such principles are not observed at all. If we ask for further equipment, the ward staff avoids doing so because they have nothing left. We are confused about whether to assign some tasks to students or not. I do not know what will happen if we work like this*.”

#### CNE under fears

Due to the nature of COVID-19, multiple fears have been raised in educators and students, particularly about the possibility of contracting the disease and transmitting it to others. By prioritizing student health conditions, the educators also sought to avoid contact with patients with suspected or confirmed COVID-19, thereby minimizing the risks of students and themselves becoming infected and transmitting the disease to their families. Participant no. 10 said that:

“*At the onset of the disease outbreak, the media reported daily on the high death toll and its rapid transmission. Cyberspace was also replete with many unknowns and much controversy about the virus. We were really scared.*”

Participant no. 1 also added that:

“*There is a lot of concern for our health and our family. When we attend the internship courses, we feel anxious that we may transfer the disease to our family, so we do not approach the patients very much.*”

During their clinical education, the students experienced a number of fears about personal protective equipment. They also used non-standard equipment and sometimes faced shortages. For example, according to participant no. 13:

“*We faced a lack of equipment at the onset of the pandemic, that is, the priority was with the hospital staff, and the school did not have that much equipment*.”

The educators and students also reiterated that they were afraid of contacting patients and their companions due to the fear of contracting the disease and transmitting it to the family because the suspected cases and sometimes the patients with confirmed COVID-19 had to be hospitalized in non-COVID-19 wards. They had thus tried to avoid any unnecessary physical and verbal contact with the patients and had experienced some kind of intimidating care.

Participant no. 14 also said that:

“*The nurses and our students, out of fear, only performed the patient care and treatment services. They did not communicate with the patient and their companions. This made them subconsciously move from fear toward not doing the same.*”

#### Lessons from the COVID crisis

According to the participants’ experiences, one of the opportunities encountered during CNE at the outbreak of COVID-19 was learning from the crisis. The achievements during this pandemic included some changes in the personal and professional lifestyles of students and educators; the use of other teaching methods; and the experience of integrating cyberspace with face-to-face internship courses.

According to participant no. 3:

“*Indeed, we have learned to provide training along with COVID-19. We behave normally and do research. We will not let COVID-19 disrupt everything.*”

Participant no. 11 also stated that:

“*I was trying to use simulated patients and role-playing techniques to show educational cases. I used to tell them to imagine a patient coming there with chest pain and asked what they were thinking about. I also played the role of a patient*.”

### CNE recovery

The participants’ experiences showed that the desired quality of CNE would occur with two themes of *“providing optimal* CNE *by eradicating COVID-19*″ and *“adapting* CNE *to existing conditions”.*

#### Providing optimal CNE by eradicating COVID-19

In this respect, the former theme included three sub-themes of *establishing interdisciplinary interactions to eradicate the disease*; *benefiting from experiences in other countries*, and *being vaccinated for COVID-19*. For example, participant no. 13 said:

“*In this era, the value of all disciplines has shown itself. COVID-19 has also created a strong link between basic and clinical sciences. We can thus see the importance of all disciplines of medical sciences together.*”

#### Adjustment of CNE to existing conditions

The participants’ experiences and views also reflected the importance of establishing interdisciplinary interactions in promoting disease prevention and control as well as patient care. Given the facilities in Iran and the potential of medical universities, CNE during the existing conditions was one way to enhance such training. Among the emerging sub-categories in this section was learning from the experiences of universities around the world, utilizing all available potentials, and empowering clinical educators. According to participant no. 7:

“*We need to see what strategies other countries with similar facilities have taken to improve their clinical nursing education, namely, learning and sharing experiences. Some universities did not allow for hospitalizing COVID-19 patients, and they remained mainly for training purposes. It was the same during the war.*”

As well, participant no. 12 added that:

“*We cannot treat all patients in the hospital; many of those who are in better condition can be treated at home. This is a great opportunity for community health nurses to demonstrate their abilities. The statistics also show that the older adults are more vulnerable to COVID-19, so who should help this age group? Who should teach them? This is where the geriatric nurses should enter.*”

## Discussion

The findings revealed the concepts for the overall challenges facing CNE during the COVID-19 pandemic. The analysis of the participants’ experiences also revealed that CNE in the time of the pandemic had posed both threats and opportunities. The results additionally indicated that COVID-19 had gained some experiences such as injured CNE, CNE under fear, and lessons from the COVID-19 crisis. As well, the finding reveals CNE recovery via providing optimal CNE by eradicating COVID-19 and adjustment of CNE to existing conditions based on participants’ experiences.

One of the side effects of the COVID-19 pandemic was unsatisfactory skill acquisition, which could lead to reduced quality of CNE, as illustrated in the participants’ experiences in the present study. In Casafont et al., this lack of skills could also bring about challenges after graduation [29]. Another study showed third-year medical students experienced a shortage of in-person training during the COVID-19 pandemic, so loss of skill development may occur [30]. Hence, it was necessary for hospitals and medical centers to provide systematic and regular guidelines for health care processes [31]. Another impact was multiple fears associated with clinical nursing education, as acknowledged by the study participants. In this regard, Nodine et al. reported that the COVID-19 pandemic had imposed much stress on nursing students due to their various clinical rotations and changes in their working hours [32]. So that a number of students had thought of leaving schools during the pandemic [33]. In the study by Alsoufi et al., half of the students also expressed their concerns about exposure to COVID-19 in their clinical wards [34]. In another investigation, Harries et al. believed that the most recent pandemic had induced moderate levels of stress and anxiety in medical students, and that using appropriate personal protective equipment had helped them to feel safe during clinical practices [35]. In the present study, the fear of being infected with COVID-19 was one of the concerns among the participants that had resulted in fears in terms of providing care to patients, but Harries et al. recruited nursing students who had accepted the risk of COVID-19 during clinical rotations. Of course, this difference in experiences could be traced back to the nature of the work of these two groups of students. In other words, nursing students, in comparison with other clinical counterparts, had experienced closer interactions and spent long hours in contact with patients with COVID-19, and were thus at a higher risk, which could bring more fear and apprehension to them. Based on the Gelchu study, nursing students experienced a fearful condition secondary to the fear of getting infection and transmitting it to their families [36].

The results of the present study also indicated some changes in the personal and professional lifestyles of the participants. Wallace et al. further found that the COVID-19 pandemic had made nursing students experience challenges such as changes in interpersonal relationships [37]. Another study on nursing students found that they were dissatisfied with the abrupt switch to online education [38]. This may be the source of stress for nursing students in various clinical settings [39]. Although the pandemic had brought challenges such as changes in teaching methods, they had turned into opportunities to experience new learning environments [40].

One of the themes emerging from this study was lessons from the COVID-19 crisis. In this regard, Shengxiao et al. further established that the pandemic was not merely a crisis; rather, it was an opportunity to reconstruct nursing students’ professional identity [41]. Due to the changes in educational conditions following the COVID-19 pandemic, the participants had found opportunities to experience other teaching methods as well as a combination of virtual and face-to-face clinical education and strategies such as adapting CNE to existing conditions. In one study, the use of interdisciplinary approaches to teaching could be effective in student education [42]. The findings by the Bdair had also shown that there was an opportunity for learning within a flexible and student-centered environment with repeated monitoring of the quality of care services provided to enhance this type of learning, although nursing students and faculty members had experienced challenges such as changes in learning environments [43]. However, in another study, 21.1% of the students believed that electronic learning could be utilized in clinical wards [35]. This different experience could be due to the multiple factors affecting electronic learning, such as electronic learning devices.

## Conclusion

The results of this qualitative study demonstrated that the COVID-19 pandemic had led the participants to experience new conditions, also referred to as “combined threats and opportunities. “Based on this, nursing managers and planners are advised to take great steps towards the development and improvement of CNE by considering and applying these results in their plans to overcome the challenge of CNE with effective and useful actions in the COVID-19 pandemic. Although changes in educational conditions, especially clinical ones, had reduced the quality of CNE and fear in the workplace, they had provided valuable opportunities to learn by taking advantage of interdisciplinary interactions to eradicate the disease, and rapid public vaccination on the one hand, and the use of experiences in other countries in terms of training students, empowering clinical educators, and using potential and available facilities on the other hand. Such threats could thus work for the benefit of the opportunities. Accordingly, nursing managers and planners were recommended to take big steps towards the development and improvement of CNE at present, with careful capacity planning to successfully overcome the challenges of the COVID-19 era.

Among the limitations of the present study was that only the experiences of students and educators at public universities were examined. In addition, due to the qualitative nature of the study, the possibility of hiding some feelings and experiences and forgetting others was raised.

## Data Availability

Due to the privacy of the research participants, the data generated during the current study are not publicly available, but are available from the corresponding author upon reasonable request.
